# Assessing multiple threats to seabird populations using flesh-footed shearwaters *Ardenna carneipes* on Lord Howe Island, Australia as case study

**DOI:** 10.1038/s41598-021-86702-4

**Published:** 2021-03-30

**Authors:** Chris Wilcox, Nicholas Carlile, Britta Denise Hardesty, Tim Reid

**Affiliations:** 1 Department of Planning, Industry and Environment, Parramatta, NSW 2150 Australia; 2 Institute for Applied Ecology, University of Canberra, Bruce, ACT 2617 Australia; 3 Present Address: CSIRO Oceans and Atmosphere, Hobart, TAS 7000 Australia

**Keywords:** Biological techniques, Ecology, Ecology, Environmental sciences

## Abstract

Globally, seabird populations have been in decline due to multiple threats throughout their range. Separating simultaneous pressures is challenging and can require significant amounts of data over long periods of time. We use spatial contrasts to investigate the relative importance of several drivers for the purported decline in a species listed as in decline as an example species, the Flesh-footed shearwater (*Ardenna carneipes*). On Lord Howe Island in the Tasman Sea, Australia, this seabird suffers from habitat loss due to housing development, intensive mortality in fisheries, plastic ingestion, and roadkill due to vehicular traffic on its breeding island. We repeated a quantitative survey of the population to ascertain whether the decline previously reported had continued and to evaluate the purported mortality sources (Reid et al. in PLoS ONE 8(4):e58230, 2013, Lavers et al. in Global Ecol Conserv 17:e00579, 2019). We measured burrow density, area of occurrence, occupancy and breeding success, integrating them with previous surveys using a Bayesian statistical model to generate longer term estimates of demographic rates. We used spatial patterns to test whether mortality on roads or proximity to human habitation was influencing population demographics. In contrast to predictions, we found the population had stabilised or increased. Characteristics such as burrow occupancy and breeding success showed little pattern, with weak evidence for impacts from road mortality and housing development. Such a data-rich approach is substantially more informative and can better support seabird conservation and management efforts does require more field-time and additional equipment than most contemporary surveys, the data is substantially more informative and can better clarify the results of efforts in seabird conservation and management.

## Introduction

Anthropogenic pressures on the marine environment have resulted in threats to a wide range of marine species. For instance, 28% of all seabirds are listed as globally threatened, making them the most threatened of all groups of birds^1^. Threats are from a range of sources, including commercial fisheries, ocean pollution, alien invasive predators and habitat destruction^1^.

Separating the effects of multiple threats in the case of seabirds, as in other taxa, is challenging. Conservationists have long appreciated the potential for additive, or even synergistic, effects of multiple simultaneous pressures on populations^2^. However, estimating the relative contribution of multiple threats is complicated, not to mention identifying synergistic effects among them^2^. While in some cases it is possible to use integrated population assessments to estimate the relative importance of an ensemble of mortality sources^3^, these methods require significant amounts of data over long periods of time, including both timeseries of population indices and specific demographic estimates. Moreover, uncertainty in the underlying data flows through these complex models, often leading to estimates of trends and mortality rates that are not very informative e.g.^4,5^.

An alternative to the typical time series approaches using population trend data is to use spatial patterns to investigate population drivers by comparing locations where those drivers differ. Substituting spatial patterns for temporal patterns is a commonly used approach to infer temporal dynamics, applied in contexts as diverse as estimating disease-host dynamics, ecosystem process, species interactions and drivers of housing expansion^6–9^. This space-for-time substitution approach can be successful, given that there are differences in the dynamic variables of interest across locations. While the approach is common across ecological and evolutionary fields for species that inhabit year-round terrestrial environments, it has rarely been used in the context of marine vertebrates, likely because they are often approached as “well-mixed” populations at sea, without taking into account their island-specific land-based activities.

One subtlety that is particularly relevant when considering spatial contrasts is the role of edge effects, in which a pressure acts at the margin of a population, potentially in a sub-lethal manner. While these edge effects can lead to significant population level pressure^10^, they have also been found to be sensitive indicators of population decline^11^. For seabirds, edges of colonies have been hypothesized as having important consequences for those individuals residing in colony margins^12^. However, as with other approaches to understanding pressures on populations, determining causality is difficult. Edges may be occupied by less experienced birds, resulting in lower fecundity or survival among nests at the colony edge^12^. Alternatively, edges may be in less suitable habitat and thus birds occupying those positions face additional pressures on foraging, reproduction, or survival^10^. Disentangling these endogenous factors from exogenous pressures, such as increased mortality due to pressure from housing development at a colony edge, is challenging as the resulting breeding failure at a location can be the same for both.

We use spatial contrasts to investigate the relative importance of four drivers (plastics, fisheries, habitat loss and roads) for the purported decline of flesh-footed shearwaters^13–17^, a hemisphere migrating seabird that nests in Australia. Historically, habitat losses^13,15^, and incidental catches by fishing vessels^16,17^ were estimated to have caused significant declines in the population from an estimated 20,000–40,000 pairs in Lord Howe Island in 1978. Even after the cessation of fisheries mortality and urban development, a recent report suggest the species is continuing to decline^14^, although the evidence is unclear and the population may have stabilized^18^. Potential explanations of declines suggested include ongoing effects from past urban development, mortality due to vehicle traffic, and ingestion of plastic fragments^13,19^.

In this paper, we first examine the current trends in the population, estimating whether previously documented declines continue. Second, we used spatial comparisons of demographic variables to examine if—and where—flesh-footed shearwater populations may be declining across Lord Howe Island. We compared support for the null hypothesis that poor/inexperienced breeders are more prevalent at edges with two hypotheses for anthropogenic threats: increased disturbance due to human activities near colony edges and direct mortality due to vehicular traffic (roads). In the case of poor/inexperienced breeders using the edges, we would expect to find all measured aspects of demography (burrow density, burrow occupancy, and breeding success) to be low at the colony edge. By contrast, disturbance effects should increase with proximity to roads, houses and tracks approximately equally, and the effect should be greatest during incubation when adults are most sensitive^20,21^. If road mortality is a significant factor, mortalities should be greater near roads and houses than other forms of edges and should be more pronounced during the fledging period relative to the incubation period.

## Results

### Census

**Figure 1:**
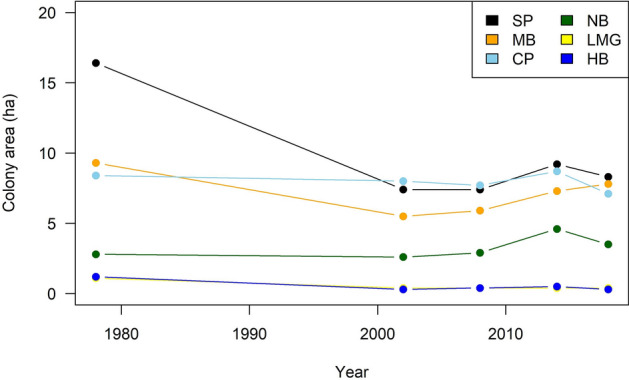
Changes in estimated flesh-footed shearwater colony areas on Lord Howe Island 1978–2018 (ha) (SP, Stevens Point; MB, Middle Beach; CP, Clear Place; NB, Neds Beach; LMBG, Little Muttonbird Ground; HB, Hunter Bay).

We estimated the total area of all Lord Howe Island six colonies as 30.7 ha (Table 1), with individual colony size ranging from 0.4 to 9.2 ha. The total area was lower than in 1978/9 (39.2 ha), higher than 2002/3 and 2008/9 (24.2 and 24.7 ha, respectively)^13^ but similar to 31.6 ha. estimated in 2017/18 by^14^. Most increases in area were in the Stevens Point and Neds Beach colonies (Fig. 1).

**Table 1:** Area, estimated flesh-footed shearwater burrow density (burrows m^−2^) and number of burrows and 95% Credible Interval for each colony on Lord Howe Island, 2014/15. Note all burrows were counted at Hunter Bay.

Colony	Area (ha)	Number of transects	Transect area (m^−2^)	No. of burrows	Mean burrow density (burrows m^−2^)	95% Credible Interval	Burrows	95% Credible Interval
Little Muttonbird Ground	0.41	2	650	25	0.039	0.028–0.062	162	114–254
Clear Place	8.76	4	2916	371	0.121	0.096–0.152	10,600	8430–13,300
Middle Beach	7.25	5	3220	359	0.105	0.084–0.130	7620	6070–9420
Neds Beach	4.61	2	1800	386	0.204	0.155–0.272	9410	7130–12,500
Stevens Point	9.21	5	3040	256	0.079	0.061–0.101	7280	5650–9320
Hunter Bay	0.50						25	
Total							35,100	31,000–39,800

**Figure 2:**
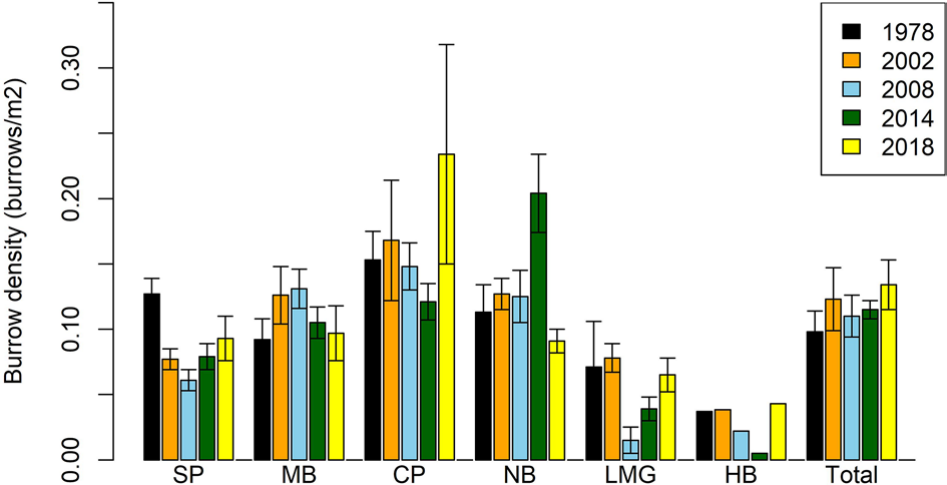
Estimated flesh-footed shearwater burrow density at each colony on Lord Howe Island in five censuses. Error bars = 1 standard deviation. (SP, Stevens Point; MB, Middle Beach; CP, Clear Place; NB, Neds Beach; LMBG, Little Muttonbird Ground; HB, Hunter Bay).

Overall, burrow density in 2014/15 was 0.115 ± 0.007 (posterior standard deviation [PSD, the standard deviation of the posterior estimate]) burrows m^−2^ (Table 1). Based on posterior estimates of the 95% credible intervals, total burrow density remained constant in all counts since 1978, although there were substantial differences for some colonies in individual years, such as an increase at Neds Beach in 2014/15 and Clear Place in 2017/2018 (Fig. 2).

**Figure 3:**
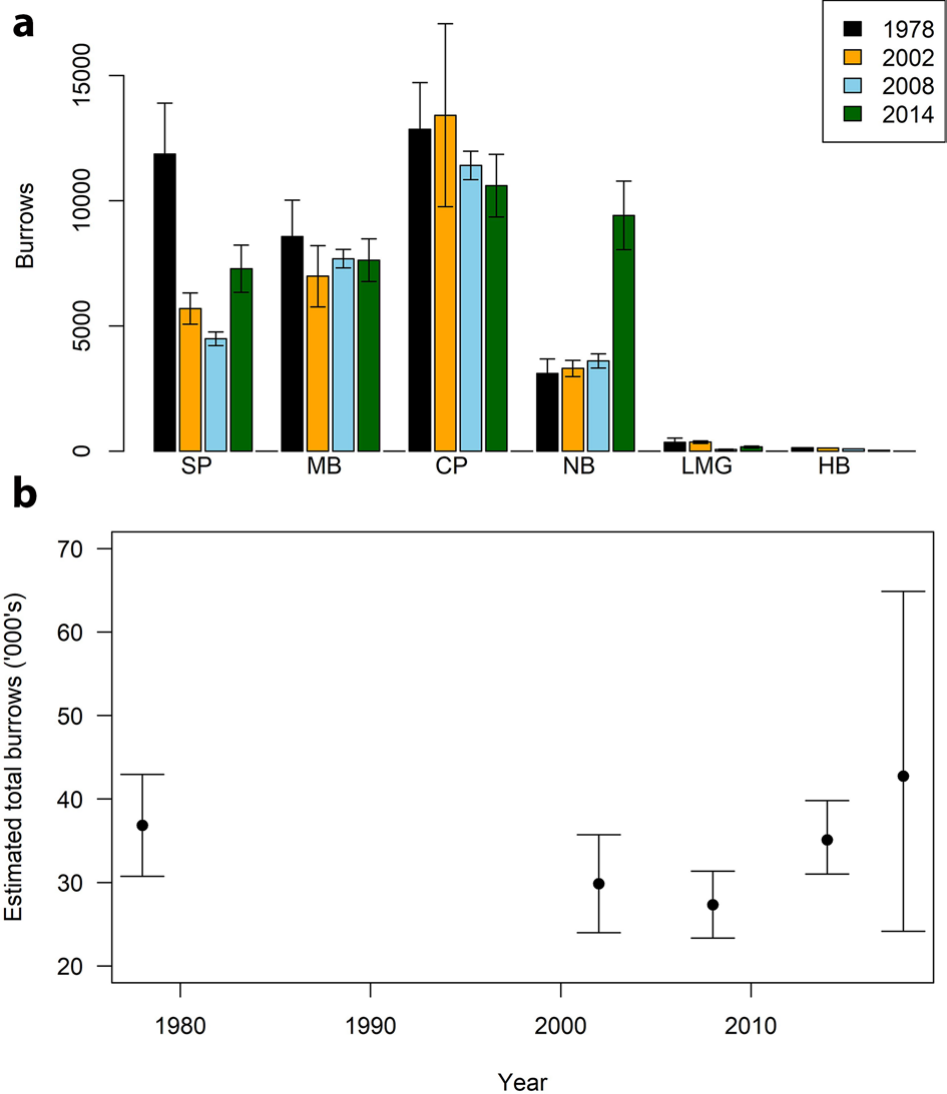
(**a**) Estimated number of flesh-footed shearwater burrows at each colony in all four years, and (**b**) estimated total burrows on Lord Howe Island.

Using burrow density and colony area values, there were an estimated 35,100 (credible limits 31,000–39,800) burrows on Lord Howe Island in 2014/15 (Table 1; Fig. 3). This was an increase on estimates for 2002/3 and 2008/9, with the increase in burrows mostly at Stephens Point and Neds Beach (Fig. 3). Most burrows were at Clear Place (31%) and Neds Beach (26%).

#### Burrow occupancy

**Figure 4:**
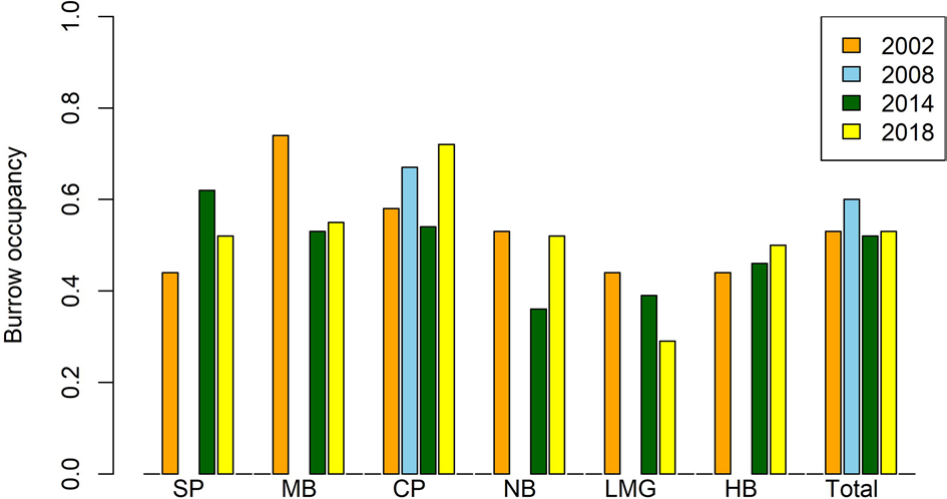
Burrow occupancy for each colony during each year. (SP, Stevens Point; MB, Middle Beach; CP, Clear Place; NB, Neds Beach; LMBG, Little Muttonbird Ground; HB, Hunter Bay).

In 2014/15, burrow occupancy was estimated at 0.52 pairs burrow^−1^ (Fig. 4). This was similar to occupancy observed in 2002/3^15^, and 2008/9^13^ (Fig. 4). Patterns of change between years were inconsistent, however, with the highest occupancy occurring at Stevens Point, and the lowest at Neds Beach and Little Muttonbird Ground (ranging from 0.4–0.6). Occupancy varied between years and between colonies with no obvious pattern (Fig. 4).

#### Breeding success

**Figure 5:**
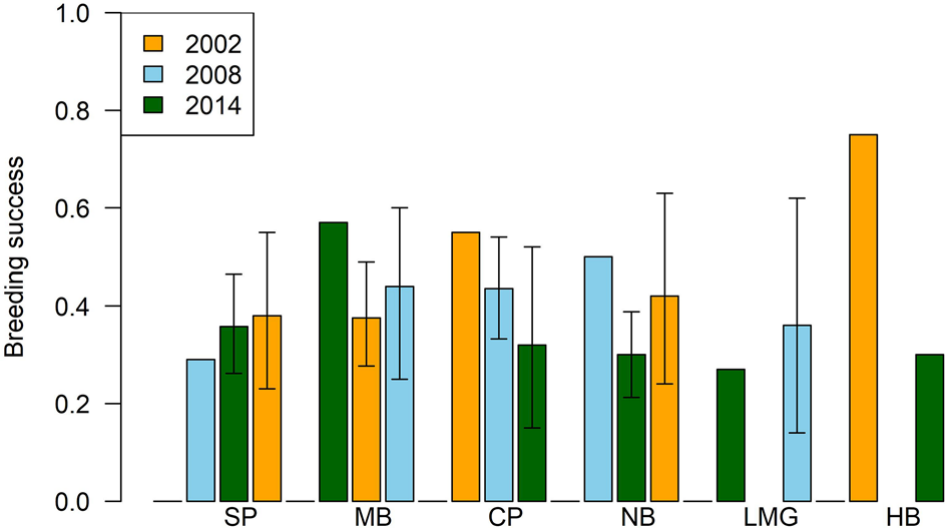
Flesh-footed shearwater breeding success in terms of chicks egg^−1^ on Lord Howe Island. (SP, Stevens Point; MB, Middle Beach; CP, Clear Place; NB, Neds Beach; LMBG, Little Muttonbird Ground; HB, Hunter Bay). (Confidence intervals for 2008 and 2014 is 95% Credible interval, no confidence intervals were indicated for 2002 by^9^, but similar methods were used to collect the data).

Breeding success was 0.38 (Posterior Standard Deviation (PSD) 0.05) chicks egg^−1^ in 2014/15 (Fig. 5), which was similar to 2008/9 (0.39) but significantly lower than 2002/3 (0.50;^13,15^. Lavers et al.^14^ observed a breeding success of 0.62 from an undisclosed single colony site. Breeding success was highest at Middle Beach and Neds Beach, and lowest at Clear Place and the small colony at Hunter Bay (Fig. 5). There was no consistent pattern between colonies among years (Fig. 5), with declines at Clear Place, increases at Stevens Point, and variability at other sites.

#### Number of chicks

**Figure 6:**
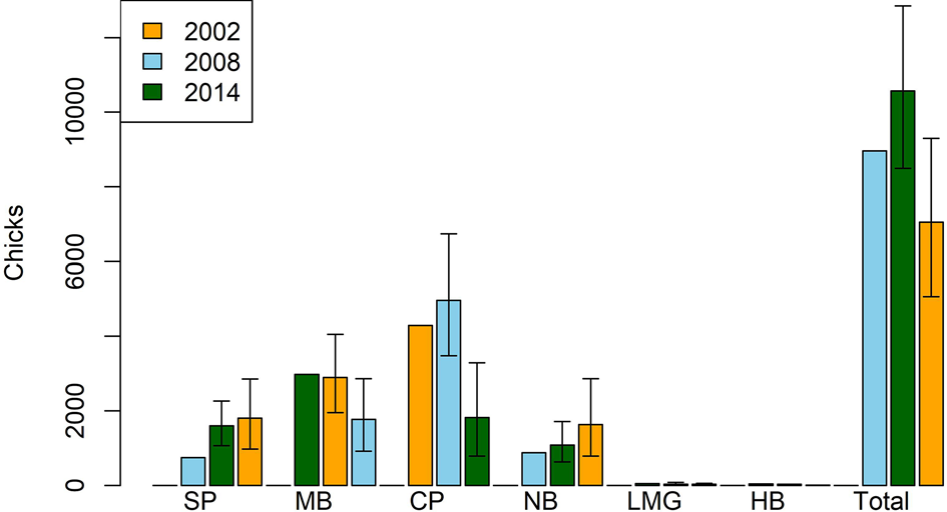
Estimated number of flesh-footed shearwater chicks produced at each colony on Lord Howe Island (SP, Stevens Point; MB, Middle Beach; CP, Clear Place; NB, Neds Beach; LMBG, Little Muttonbird Ground; HB, Hunter Bay). (Confidence intervals for 2008 and 2014 is 95% Credible interval, no confidence intervals were indicated for 2002 by^9^.

An estimated 7050 (PSD 1009) chicks were produced on Lord Howe Island in 20,014/15 (Table 2). Most chicks were produced in Clear Place and Stevens Point (26% each). This is a 34% decrease in the estimated number of chicks produced compared with 2008/9, with most of the decrease due to an apparent halving in the number of chicks produced at Clear Place (Fig. 6;^13,15^).

**Table 2:** Estimated number of chicks from each colony on Lord Howe Island, 2014–2015.

Colony	Chicks	95% Credible Interval
Little Muttonbird Ground	25	8–56
Clear Place	1820	786–3290
Middle Beach	1770	914–2860
Neds Beach	1630	786–2860
Stevens Point	1800	971–2850
Hunter Bay	4	
Total	7050	5060–9300

#### Number of breeding pairs

The number of breeding pairs in 2014/15 was estimated at 18,500 (2.5–97.5% credible limits 15,300–22,000) breeding pairs. The odds of an increase in breeding pairs since 2002 was 106% (88–126%), since 2009 was 114% (94–135%). This gives an apparent annual rate of increase in the number of breeding pairs since 2002 of 0.4% (− 1.0 to 1.8%) and since 2009 of 2.1% (− 1.0 to 5.2). Other studies reported 22,654 (8156–37,909) breeding pairs in 2017/2018 possibly indicating this possible recovery is continuing^14^.

### Road mortality

Three carcases were located over 81 transects. This is a density of 0.93 carcasses/1000 m^2^ (credible limit 0.19–2.26). From this, we estimated that 45 birds were killed on Lord Howe Island roads during 2014/15 (95% credible limit 9–110).

### Spatial modelling of burrow habitat and occupancy

#### Breeding success

**Figure 7:**
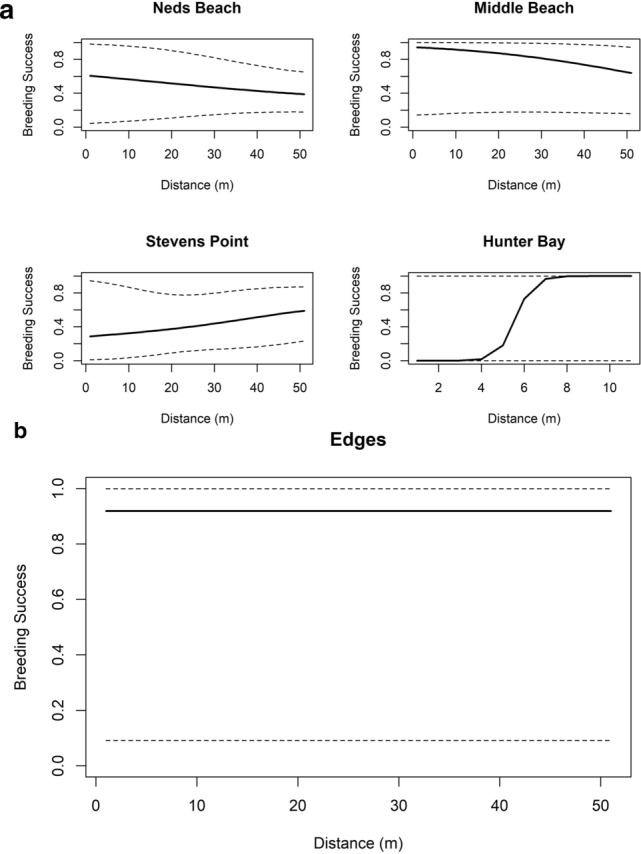
Predicted breeding success by main effects of best model in Table 3; (**a**) distance (in metres) to roads/colony; and (**b**) distance to edges (in metres).

Two models were retained for breeding success (with AIC weighting of 0.984; Table 3). Distance from roads and other edges (without roads, houses or tracks) were found to affect breeding success. Of these, when the weight was summed for models containing each variable, distance to roads had the most importance. The model with the lowest AICc accounted for 9% of variance in the data, based on a deviance comparison. The full model suggested that breeding success changed with distance from roads and colony edges, but it varied between colonies (Fig. 7).

**Table 3:** Best models of flesh-footed shearwater breeding success on Lord Howe Island, with their degrees of freedom, Log-likelihood, AICc and delta AICc, and weighting from model averaging.

Model	*df*	logLik	AICc	Delta	Weight
S_1_(roads) + Colony + S_2_(roads, by = as.factor(Colony))	19	− 271.29	583.12	0	0.708
S_3_(edges)	9	− 282.46	584.89	1.772	0.292

Terms s(…) indicated a smoothed GAM term.

#### Burrow occupancy

A null model of burrow occupancy (constant average occupancy) fitted the data best, indicating there was no significant spatial pattern with respect to edges or potential mortality sources.

#### Burrow density

**Figure 8:**
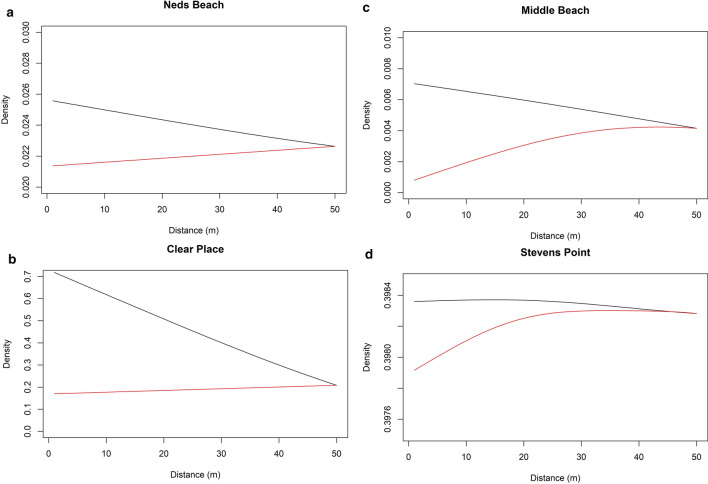
Predictions of burrow density at progressive distances (up to 50 m) while holding all other variables constant. Plots for each of the 4 main colonies; black = roads, red = houses.

Burrow density was best fit by a full model, with all environmental variables affecting burrow density (Supplementary Tables 2 and 3). This model explained 92% of deviance. Burrow density decreased with increasing distance from roads (Fig. 8). Burrow density increased away from houses at all colonies (Fig. 8). Changes in burrow density away from edges and tracks varied between colonies (Supplementary Fig. 1).

## Discussion

We investigated the population trends of flesh-footed shearwaters on Lord Howe Island in light of previous evidence of declines^13,15^ and given the existence of multiple stressors likely to affect the population^22–24^, including habitat destruction, disturbance, fisheries mortality and pollution. In contrast to our expectation, we found the total number of breeding pairs of flesh-footed shearwaters on Lord Howe has been stable or slightly increasing since 2008/9. This suggests either there has been some rebound in the population, or simply variability in the estimates, or both. After previous reported declines^13,15^, both the colony area and the number of burrows has increased. However, the apparent direction of the effect of other demographic indicators has been variable. Our results suggest that over the period of decline and recovery, there were only limited effects of human impacts on colony demographics such as burrow occupancy or density. We are not questioning the validity of earlier findings, rather acknowledging that the population may be rebounding or highlighting the dynamism in the system. Furthermore, we are aware of public awareness campaigns aimed at reducing road traffic speed. It is possible that the increase in burrow numbers reflects the success of such campaigns.

We modified common seabird colony surveys in two ways, which could be broadly useful for other efforts. First, we recorded GPS positions for every burrow included in our study. This allowed us to use spatial models to evaluate the support for various hypotheses we have suggested regarding disturbance and vehicle strikes. Second, we oriented our survey transects perpendicular to colony edges, allowing us to create a much more representative survey dataset for habitat use and breeding success. Incorporating these approaches into long-term monitoring programs can provide significant benefits in assessing threats from multiple causes and in evaluating the effectiveness of interventions. While this approach does require more effort and additional equipment, by increasing the quantity and quality of data, it can be substantially more informative on an increased number of questions, for a marginal increase in effort.

Overall, the estimates of population size that depend on colony area suggest an increase in the flesh-foot shearwater population, while the metrics that depend only on the repeat transect surveys (burrow density and occupancy) suggest they are variable among years. Given the difficulties with measuring colony edges for a burrowing species in a habitat with mixed land use, dense and variable vegetation, and changes in land access, we suggest that the area-based population size should be treated with caution. Burrow density and occupancy are likely less sensitive to population change, as they are measured along transects centrally located in colonies, where change will likely be slowest^11^. Measurements along transects used in surveys in different years will be less subjected to measurement error, but as they cover the centre and edges of colonies, these surveys should still be relatively sensitive to changes.

Breeding success on Lord Howe Island has been quite variable over 16 years, ranging from 39–62%. In other breeding colonies in Australia, such as Woody Island in Western Australia, breeding success over four years was 40% to 53%^25^. With five censuses in total across a 42 year timespan (and three for breeding success and productivity), it remains difficult to draw conclusions^26^. This highlights issues for long lived species with long generation times meaning that fewer surveys or shorter time scales may highlight temporary or intermittent highs or lows in the system. This points to the value of long term monitoring efforts to understand patterns through space and time, which is particularly important as stressors on the system/taxa are undoubtedly variable (change through space and time).

Mortality of shearwaters on roads was estimated at 125 birds/year during 2008/2009^13^, which is more than double that estimated for 2013/14 (45 birds/year). There was a considerable campaign to educate local drivers after the 2008/2009 estimate (H. Bower pers. comm.), thus the decrease may be due to decreased mortality. Alternatively, this may have caused drivers to stop and retrieve carcases and then hide them as a response to the campaign, as there was some evidence for this (H. Bower pers. comm.).

We predicted that with high road mortality recorded in the past^10,12,13^, there should be decreased breeding success and burrow occupancy near roads^27^. We found only weak evidence for any effects on breeding success due to roads or other edges. If anything, there was a slight increase in burrow density and breeding success with proximity to roads. The relationship not matching our prior expectations could be due to two mechanisms. Road mortality may be insufficient to affect nearby burrows, and so having no affect on the population^13^. Alternatively, our early incubation stage occupancy surveys may have been too early to detect the loss of adults near roads, as this mortality will accumulate as the breeding season progresses. There was no effect of roads on burrow occupancy. It is possible that burrows near roads are rapidly re-colonized by new pairs each year if the previous years’ occupants failed due to road mortality of one or both adults. This is potentially likely if the density of burrows is at or near saturation, or burrows near roads are attractive due to features such as soil characteristics (e.g. soft soil that is suited to burrowing), or availability of take-off/landing space. Breeding success was expected to show a stronger pattern with respect to other characters, as it is affected by cumulative mortality over the whole breeding season, which should increase the strength of the mortality signal. Breeding success is purely a within season metric, unlike burrow occupancy, which is affected by both within and between season dynamics^28^.

We predicted that if road mortality was a major factor affecting the population, we should find a negative relationship between proximity to roads and breeding success, burrow occupancy, and potentially burrow density^10,12,29^. Moreover, we predicted the strength of these relationships should be burrow density < burrow occupancy < breeding success. If road mortality is the driver, instead of just general disturbance, these patterns should be stronger at colony edges where roads are present than those adjoining trails, houses, or natural vegetation. In contrast, the results we observed were equivocal^30^. While the measured edge effects had strongest effect on burrow density, they had little or no effect on occupancy or success. In addition, the road effect was in the opposite direction to that expected. This supports the idea that the road mortality has declined, at least in recent times. Perhaps this is due to increased awareness of locals on the island, in association with outreach campaigns targeted to reduce traffic speeds on the road, particularly during the breeding season (N Carlile, pers. obs).

## Conclusion

The number of breeding pairs of flesh-footed shearwaters on Lord Howe Island, which had previously been suggested to be declining^14^, appears to have stabilised or increased in the period 2009–2017. Road mortality has previously been estimated to be substantial^13^. However, it appears that the threat of road mortality has been reduced, likely due to island-wide awareness raising campaigns and the associated support from the resident island population. While there is still some evidence that some demographic variables such as breeding success or burrow density decline near potential human impacts such as roads on the colonies, it is generally weak.

These results for the Lord Howe Island population suggest previously documented threats are abating. Habitat loss was previously significant^15^, but is no longer occurring. Concordantly, the colony area for flesh-footed shearwaters on the island is now increasing. Road mortality has apparently been curtailed. Bycatch in longline fisheries has previously been documented as significant^16,31^, but due to recent fisheries management efforts, has been much reduced^32^. The population estimate here suggests the population is increasing. There has been some lag in recognizing this recovery, with studies suggesting a continued decline e.g.^14^. As in any other science, applied ecological research can suffer from confirmation bias, which can undermine the credibility of research results with policy makers and the public (e.g.^33^). This issue is particularly problematic in contexts where there are multiple threats, with complex interactions and long lag times. Such contexts call for careful examination of evidence, and integrative methods that allow meta-analysis across studies.

## Materials and methods

Lord Howe Island is a small (1455 ha) island of volcanic origin located approximately 495 km east of Australia^34^. The island is crescent shaped with extensive shallow coral reefs enclosed in a lagoon on the western side. The southern end of the island is dominated by remnant volcanic plugs rising to 875 m. The northern hills rise to 209 m, and together with nearby outer islands, are part of the remains of a larger older caldera^35^. These are separated by an area of lowlands derived from coral-derived calcarinite^36^. Much of this lowland area has been developed for agriculture and human settlement. For details of vegetation communities on the island see^36^.

**Figure 9:**
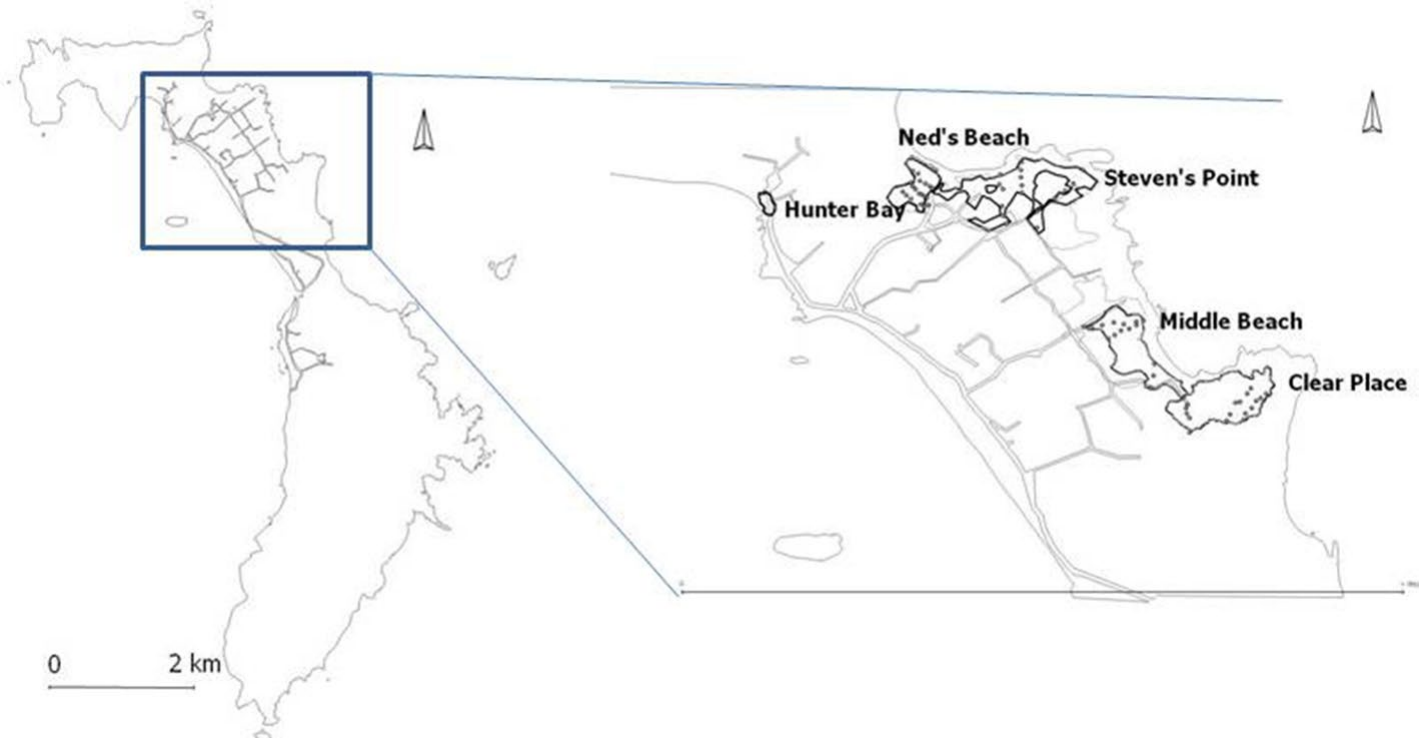
Location of colonies in Lord Howe Island. Map created using R open source software^44–46^ using data supplied by authors and Lord Howe Island Board.

The flesh-footed shearwater is a medium sized (550–750 g) shearwater^37^ which, like other procellariiforms lays a single egg in a burrow^38^. They breed on a number of islands in the southern hemisphere, around New Zealand, in southern Western Australia, on Lord Howe Island, on Phillip Island off Norfolk Island and on Íle Saint-Paul in the Indian Ocean, and migrate to the northern hemisphere for the Austral winter, concentrating in the Arabian Sea^39^ and in the North Pacific^37,40^ (Carlile unpub. data). There is evidence of genetic divergence between populations in the Pacific Ocean and those in southern Western Australia and the Indian Ocean^41^. On Lord Howe Island the flesh-footed shearwater breeds in lowland areas, predominantly in sandy soil under palm forests on the eastern side of the island. There are currently five discrete colonies on the eastern side (Neds Beach, Stevens Point, Middle Beach, Clear Place and Little Muttonbird Ground), with a small number also breeding in a single colony on the western side (Hunter Bay) (Fig. 9). Flesh-footed shearwaters are not known to breed on any of Lord Howe’s offshore islands^42,43^.

### Burrow transects

We used eighteen established strip transects across five of the colonies to evaluate burrow density in colonies. These transects were used in previous studies^13,15^, and totalled 2.9 km in length and approximately 7% of the colony area. A later published study has used the same transects, and where appropriate, these results have been incorporated into our analysis^14^. Transects were evenly separated and oriented perpendicular to the longest axis of the colony, passing between colony edges through the centre of the colony. All burrows within two meters of either side of each transect were counted, and their perpendicular distance from the transect was recorded. Detection of burrows was consistent over the entire width of each transect. All burrows were checked if they were large enough for a flesh-footed shearwater to enter (entrance > 10 cm high) and deeper than 40 cm^26^.

Burrow surveys took place during two periods; between 12–18 December 2014 and 11–13 April 2015. During December, 250 burrows (50 per colony) were individually marked and checked using a burrowscope to determine if the burrow was occupied by an adult with an egg. In April 2015, these burrows were similarly reexamined to determine which of them contained a near fledged bird. Burrows were examined using a burrow scope (Peep-A-Roo: Sandpiper Technologies) consisting of a 4 m, light sensitive, camera probe illuminated by infrared LED’s with the image hard-wired to the operator through video goggles. The position of all occupied burrows was taken with a Garmin GPS unit accurate to < 5 m. This work was carried out in accordance with the New South Wales Office of Environment and Heritage (OEH) Scientific license SL100668 and OEH ethics approval number 021028/02, and all methods and protocols were conducted in accordance with these. No animals or plants were manipulated or experimented on.

We incorporated existing data into our analysis where possible. Data on area, burrow density occupancy and success were available for most surveyed years. However only area and burrow density were available for 1978/1979, and complete data for 2008/2009 was only collected at Clear Place.

### Census of breeding colonies

An island-wide census was conducted in 2014/15, using methods used in other censuses in 1978/1979, 2002/2003, 2008/2009 and 2017/2018^13–15^. The island was exhaustively surveyed to identify new locations for breeding birds. The area of each colony was measured by walking the perimeter with a hand held GPS (Magellan Professional Mobile Mapper 6) and measuring the area of the resultant polygon.

Burrow density was estimated using the counts of suitable burrows recorded during the transect surveys (except Hunter Bay where it was possible to count all burrows). For census purposes, transects were divided into 10 m lengths, giving counts of burrows in 40 m^2^ sections for analysis. The data were treated as count data, with the total number of burrows per section as the response variable for statistical analysis. Fitted values for the number of burrows per transect section were then used to estimate standardized burrow density.

Estimates of colony characteristics were the same as those used by^13^. In brief, the number of burrows in each colony was estimated by calculating the mean and Posterior Standard Deviation (PSD) density of burrows in each colony from the transect counts, and multiplying that by the colony area (Fig. 1). The credible intervals were calculated from the posterior. We estimate the total number of breeding pairs in each colony as the number of burrows multiplied by the occupancy rate^15^.

### Breeding success

The counts of birds on eggs from the December survey and burrows with chicks from the April survey were used to estimate breeding success. Both of these measures were assumed to come from a Bernoulli distribution, as each involves only two states (success or failure). From these we can derive the breeding success (eggs producing a fledgling) and burrow occupancy (burrows with a breeding pair). Burrow productivity (fledglings per burrow) is estimated as the product of these two quantities. Some burrows were found in April to contain chicks where eggs or incubating adults had been undetected in December; this was adjusted for using previously outlined methods^15^.

We assumed that as fledging occurs for this species in late April or early May^15^, the observed advanced chicks were likely to survive to fledging and the counts would therefore provide a reasonable estimate of burrow productivity. We estimated the number of fledglings produced as the product of the number of burrows in each colony, and the productivity of that colony.

### Road mortality

On 19 December 2014, transects were established on both edges of all roads in areas of known or suspected breeding habitat. In total 80 transects that were four meters wide by ten metres in length at ten metre intervals were established perpendicular to roads and searched to estimate the numbers of shearwaters killed on the roads from the seasons commencement until the early incubation period. Additionally, a single six metre transect was used where the colony was only six metre wide at that point. Carcasses were uniformly detectable two metres to either side of transects, giving an area of coverage of 3224 m^−2^. We maintained the previously reported assumptions of prevalence of road kills across the colony and carcass longevity^13^. Transects were re-counted on 4 April 2015.

### Modelling burrow habitat and occupancy

Burrows were modelled in relation to their environment by locating their distance from features hypothesised to be of interest (e.g. roads, houses, colony edges), as well as the class of habitat they were situated in (vegetation, geology). Three characteristics of burrows were modelled: burrow density, burrow occupancy and breeding success. GIS overlays of six environmental variables supplied by the Lord Howe Island Board were used for examining factors affecting burrow characteristics (geology, vegetation type, and distance to each of roads, houses, tracks and edges). Distances to colony edges were based on a polygon layer, created from the colony boundary GPS data collected to measure colony size.

Breeding success and burrow occupancy were modelled using logistic regression generalised additive models implemented in the mgcv package in R software^44,45^. For analysing breeding success, extra data were available for four colonies collected during 2008. These were added due to the limited number of data for breeding success using only 2014, and accounted for in the statistical model with a year term. We used AICc to weight the models by the quality and estimate the values for the model terms based on our hypothesized drivers (distance to roads, houses, tracks and edges) of distribution and demography^46,47^. Model averaging was performed using the R package MuMIn^47^.

Model averaging was made on a series of models based on our hypothesized environmental drivers (distance to roads, houses, tracks and edges), allowing for colony differences, and exploring potential explanations for mortality (initially 24 models). The models included main effects of each environmental variable and with an interaction term by Colony, as it was hypothesised that success would vary between the colonies. We explored both spline and linear relationships in order to examine if there were linear or non-linear effects (Supplementary Table 1). The initial 24 models were reduced to 18, as some models were identical for spline and linear structures. For model averaging, models with an AIC within two of the best model were retained.

## Supplementary Information

Supplementary Information.
