# Supplementary material for: Assessing multiple threats to seabird populations using flesh-footed shearwaters Ardenna carneipes on Lord Howe Island, Australia as case study
Source: Sci Rep. 2021 Mar 30;11:7196. doi: 10.1038/s41598-021-86702-4 (PMC8009916; doi:10.1038/s41598-021-86702-4)
Supplement: Supplementary file 1 — Supplementary Information. [file 41598_2021_86702_MOESM1_ESM.docx]

Assessing multiple threats to seabird populations using flesh-footed shearwaters *Ardenna carneipes* on Lord Howe Island, Australia as case study.

Authors

Chris Wilcox CSIRO Oceans and Atmosphere, Hobart, Tas 7000

Nicholas Carlile, Department of Planning, Industry and Environment, Parramatta, NSW 2150

Britta Denise Hardesty, CSIRO Oceans and Atmosphere, Hobart, Tas 7000

Tim Reid^*#!^ Institute for Applied Ecology, University of Canberra, Bruce, ACT, 2617

^*^corresponding author

^#^Current address: CSIRO Oceans and Atmosphere, Hobart, Tas 7000

^!^Correspondence to Tim.Reid@csiro.au

Supplementary Material

Model 1: Occupancy ~ 1

Model 2: Occupancy ~ roads

Model 3: Occupancy ~ houses

Model 4: Occupancy ~ edges

Model 5: Occupancy ~ habitat

Model 6: Occupancy ~ geology

Model 7: Occupancy ~ Colony

Model 8: Occupancy ~ Colony * roads

Model 9: Occupancy ~ Colony * houses

Model 10: Occupancy ~ Colony * edges

Model 11: Occupancy ~ Colony * habitat

Model 12: Occupancy ~ Colony * geology

Model 13: Occupancy ~ s(roads)

Model 14: Occupancy ~ s(houses)

Model 15: Occupancy ~ s(edges)

Model 16: Occupancy ~ s(roads) + Colony + s(roads, by = as.factor(Colony))

Model 17: Occupancy ~ s(houses) + Colony + s(houses, by = as.factor(Colony))

Model 18: Occupancy ~ s(edges) + Colony + s(edges, by = as.factor(Colony))

Supplementary Table 1. Models used in model averaging of burrow habitat detail. Each of the models were tested for burrow density, burrow occupancy, and breeding success.

| Coefficient | df | F | p-value |
| --- | --- | --- | --- |
| Geology | 2 | 15.26 | < 0.001 |
| Habitat | 5 | 7.15 | < 0.001 |
| Colony | 4 | 5.71 | < 0.001 |
| Geology:Colony | 4 | 5.52 | < 0.001 |
| Alliance:Colony | 9 | 5.21 | < 0.001 |

Supplementary Table 2. Categorical terms for the full model for flesh-footed shearwater burrow density on Lord Howe Island.

.

| Coefficient | edf | F | p-value |
| --- | --- | --- | --- |
| Longitude*Latitude | 28.18 | 67.42 | < 2e-16 |
| Roads*Colony |  |  |  |
| Clear Place | 1.77 | 28.50 | < 0.001 |
| Little Mutton Bird Ground | 0.84 | 0.34 | 0.59 |
| Middle Beach | 0.84 | 20.35 | < 0.001 |
| Neds Beach | 0.83 | 10.73 | < 0.001 |
| Steves Colony | 1.80 | 36.58 | < 0.001 |
| Houses*Colony |  |  |  |
| Clear Place | 0.92 | 35.86 | < 0.001 |
| Little Mutton Bird Ground | 0.83 | 1.63 | 0.24 |
| Middle Beach | 0.83 | 2.67 | 0.14 |
| Neds Beach | 0.96 | 5.95 | 0.06 |
| Steves Colony | 0.86 | 3.28 | 0.09 |
| Edges*Colony |  |  |  |
| Clear Place | 1.82 | 35.24 | < 0.001 |
| Little Mutton Bird Ground | 1.79 | 8.11 | < 0.001 |
| Middle Beach | 0.84 | 18.78 | < 0.001 |
| Neds Beach | 1.76 | 6.74 | < 0.001 |
| Steves Colony | 1.83 | 31.67 | < 0.001 |
| Tracks*Colony |  |  |  |
| Clear Place | 1.12 | 0.45 | 0.37 |
| Little Mutton Bird Ground | 0.94 | 1.96 | 0.28 |
| Middle Beach | 1.82 | 17.49 | < 0.001 |
| Neds Beach | 1.12 | 0.82 | 0.47 |
| Steves Colony | 1.83 | 33.16 | < 0.001 |
| Roads | 0.83 | 26.30 | < 0.001 |
| Houses | 0.99 | 3.02 | 0.14 |
| Tracks | 0.83 | 0.48 | 0.53 |
| Edges | 0.84 | 16.35 | < 0.001 |

Supplementary Table 3. Smoothed terms for the full model for flesh-footed shearwater burrow density on Lord Howe Island.. edf=estimated degrees of freedom

Supplementary Fig 1. Change in burrow density with increasing distance (metres) from edges and tracks; a. Neds Beach; b. Clear Place; c. Middle Beach; d. Stevens Point (black=edges; red=tracks).

a. b.


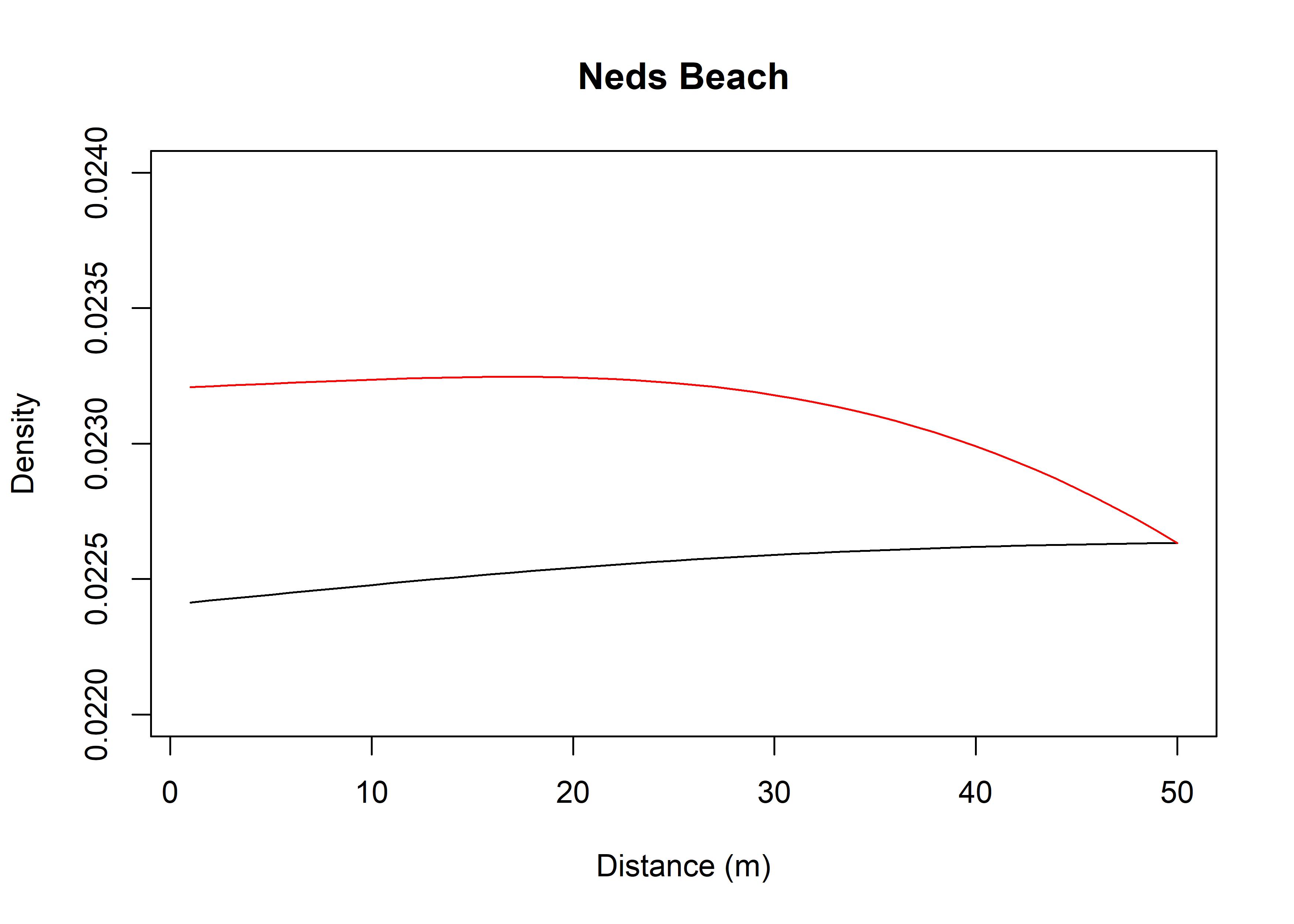

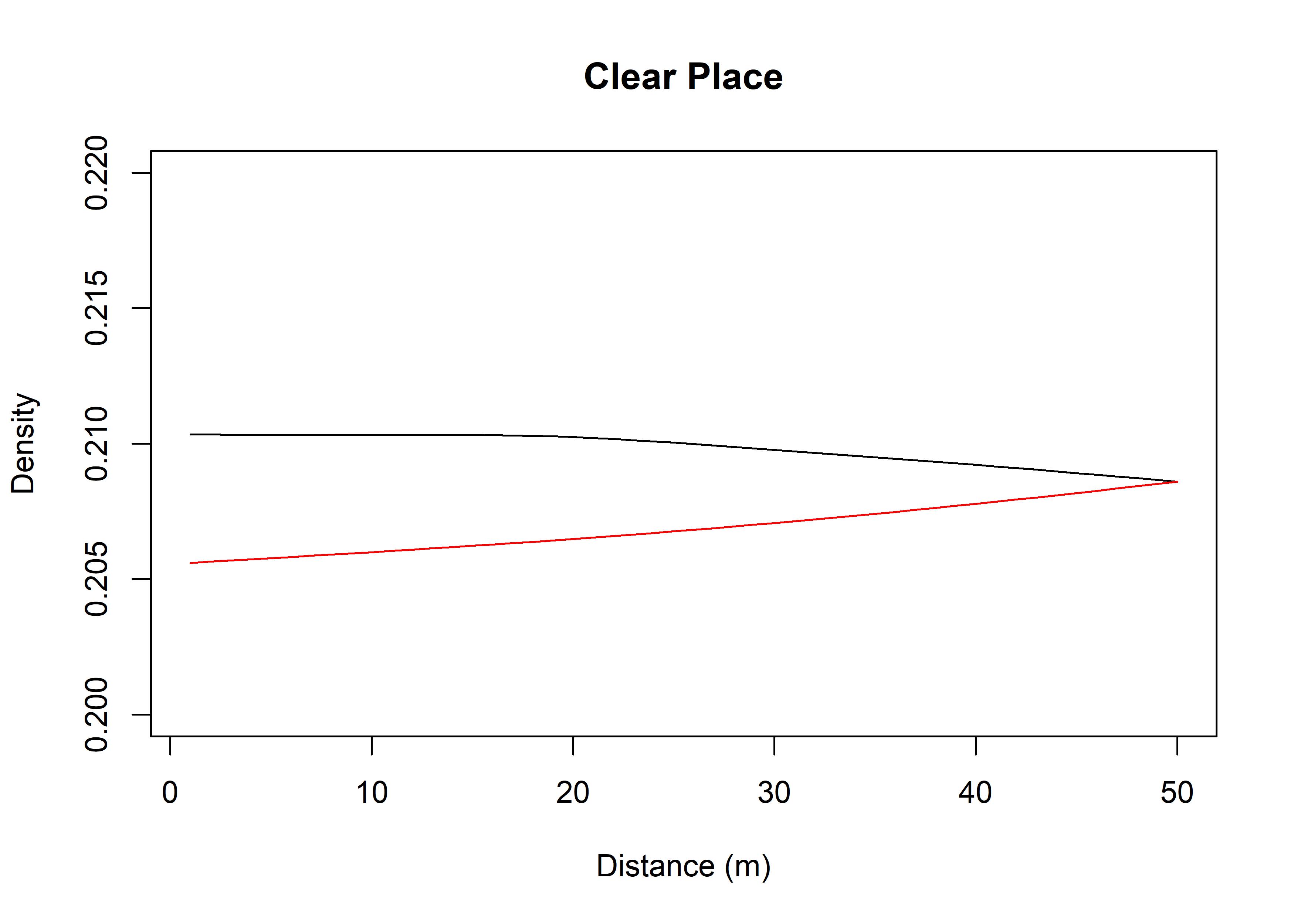


c. d.


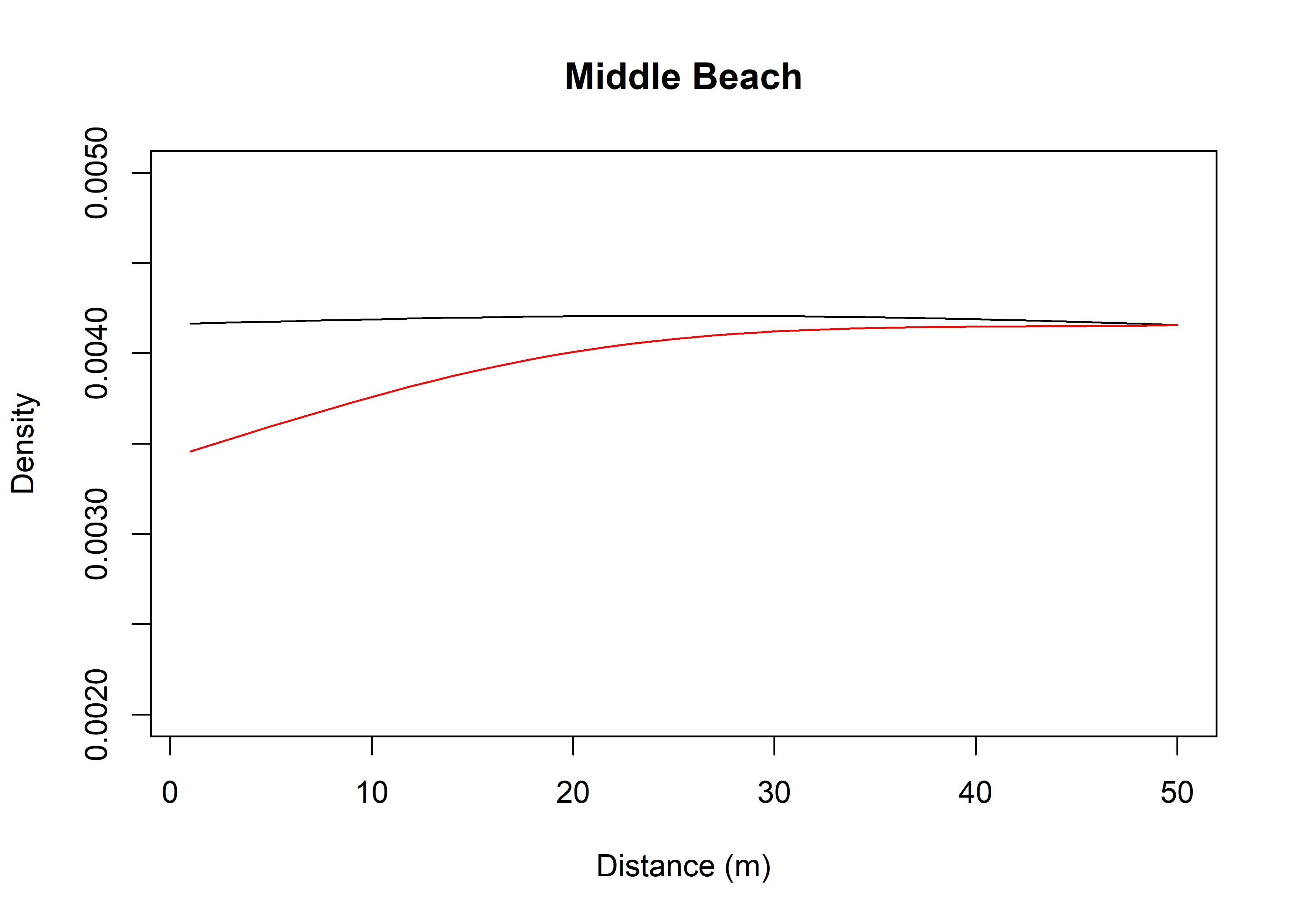

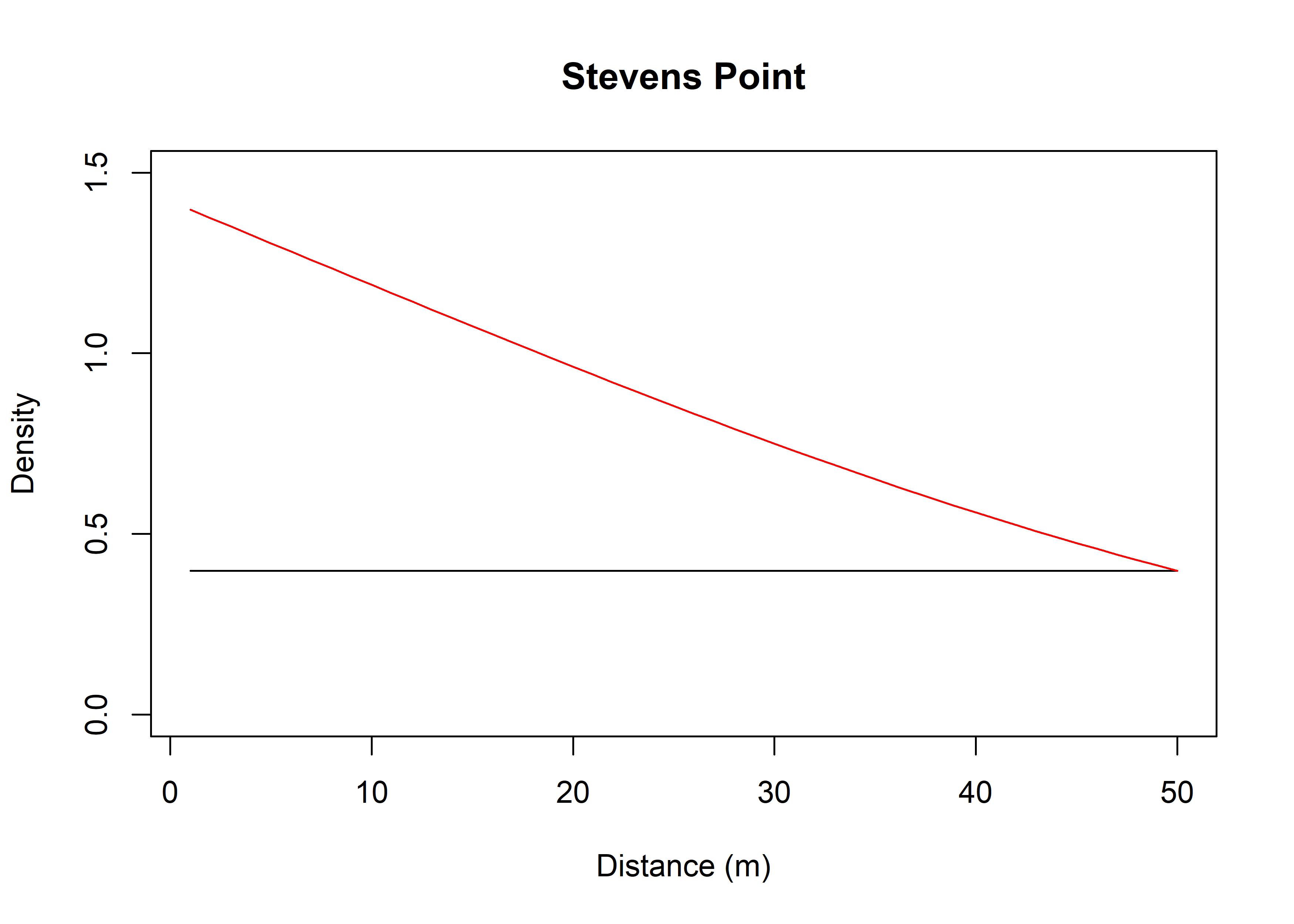


b.
